# Preparation of Ce-MnO_x_ Composite Oxides via Coprecipitation and Their Catalytic Performance for CO Oxidation

**DOI:** 10.3390/nano13152158

**Published:** 2023-07-25

**Authors:** Junsheng Yang, Jie Li, Jiangang Kang, Wenkang Liu, Yijian Kuang, Hua Tan, Zhensen Yu, Liu Yang, Xuejin Yang, Kui Yu, Yiquan Fan

**Affiliations:** 1 College of Mechanical Engineering, Wuhan Polytechnic University, Wuhan 430048, China; yangjunsheng2008@163.com (J.Y.); lj820680639@163.com (J.L.); a2300120524@163.com (W.L.); kuangyijian1999@163.com (Y.K.); yuzhensen2002@163.com (Z.Y.); yangliuvictry@163.com (L.Y.); yangxuejin.2007@163.com (X.Y.); 2 Zhongye Changtian International Engineering Co., Ltd., Changsha 410205, China; csukjg@126.com; 3 State Key Laboratory of Material Processing and Die & Mould Technology, Wuhan 430074, China; hua_tan@hust.edu.cn; 4 School of Materials Science and Engineering, Huazhong University of Science and Technology, Wuhan 430074, China

**Keywords:** coprecipitation method, CO catalytic oxidation, CO catalytic performance, Ce-MnO_x_ catalysts

## Abstract

Ce-MnO_x_ composite oxide catalysts with different proportions were prepared using the coprecipitation method, and the CO-removal ability of the catalysts with the tested temperature range of 60–140 °C was investigated systematically. The effect of Ce and Mn ratios on the catalytic oxidation performance of CO was investigated using X-ray diffraction (XRD), X-ray energy dispersive spectroscopy (EDS), scanning electron microscopy (SEM), H_2_ temperature programmed reduction (H_2_-TPR), CO-temperature programmed desorption (CO-TPD), and in situ infrared spectra. The experimental results reveal that under the same test conditions, the CO conversion rate of pure Mn_3_O_4_ reaches 95.4% at 170 °C. Additionally, at 140 °C, the Ce-MnO_x_ series composite oxide catalyst converts CO at a rate of over 96%, outperforming single-phase Mn_3_O_4_ in terms of catalytic performance. With the decrement in Ce content, the performance of Ce-MnO_x_ series composite oxide catalysts first increase and then decrease. The Ce MnO_x_ catalyst behaves best when Ce:Mn = 1:1, with a CO conversion rate of 99.96% at 140 °C and 91.98% at 100 °C.

## 1. Introduction

A fresh focus on CO emissions has evolved as the issue of air pollution garners increasing attention in many countries, and strict emission standards have also been proposed in plans. In East Asia, the main source of CO is industrial sources. However, the CO concentration in East Asia decreased with an annual trend of 0.41 ± 0.09% between 2005 and 2016, with China alone accounting for 84% of the CO reduction [1]. In Pakistan, carbon monoxide mainly comes from industrial production and transportation, with car emissions, fossil fuels, and biomass combustion being the main sources of CO, and pollutant levels in congested areas are higher than in open areas [2]. Catalytic oxidation [3], in which CO is oxidized to CO_2_ by a catalyst under certain conditions, is one of the most effective ways to remove CO in the face of these extremely stringent criteria and considerable emissions.

At the moment, the catalyst system used for CO oxidation can be categorized as noble metal catalysts (such as Pt, Au, and Pd) [4,5,6], as well as non-noble metal catalysts represented by metal oxides and composite metal oxide catalysts [7,8,9]. Non-noble metal catalysts have drawn a lot of interest in comparison to noble metal catalysts because of their low cost and abundant raw material sources. Manganese is frequently utilized in the field of catalysis because it possesses a variety of oxides in different valence states and a good redox capability [10,11,12]. Mobini et al. [13] synthesized and studied the effect of Mn supported on different metal oxides on CO low-temperature oxidation reaction using the coprecipitation method and sol-gel method. They emphasized that 20 wt% Mn/CeO_2_ has high dispersity, proper reducibility, and the maximum concentration of active site. The temperature corresponding to 50% CO conversion (T_50_) is about 142 °C. By using the citric acid-nitrate spontaneous combustion method, Mahnaz et al. [14] prepared LaMnO_3_ catalysts with calcination temperatures of 600 °C and 900 °C and discovered that the CO conversion rate reached 90% at 162 °C and 228 °C, respectively. Cerium oxide exhibits remarkable catalytic performance due to its fluorite-type structure, good oxygen storage capacity, and ability to undergo reversible transformation into Ce^3+^ and Ce^4+^ [15]. Venkataswamy et al. [16] employed the hydrothermal method, coprecipitation method, and gel sol method to fabricate catalysts with a fixed Ce:Mn ratio of 0.7:0.3. The result revealed that the as-prepared Ce_0.7_Mn_0.3_O_2−δ_ catalyst by hydrothermal method showed the best catalytic activity, the temperature required for T_90_ was 132 °C, while that prepared by coprecipitation method and gel-sol method were 158 °C and 216 °C, respectively. To prepare a CO catalyst with a fixed cerium manganese ratio of 1:8, Ye et al. [17] employed citrate sol-gel (C), hydrothermal (H), and hydrothermal-citrate complexation (CH) methods, with T_90_ at 110 °C, CH-1:8 achieving the best CO-conversion rate, followed by H-1:8 (T_90_ = 142 °C) and C-1:8 (T_90_ = 205 °C). Zhang et al. [18] introduced monodisperse Mn-doped CeO_2_ nanoparticles using a template-free solvothermal method and found that the CeO_2_ nanoparticles prepared via Mn doping had higher CO catalytic oxidation activity than the pure CeO_2_ prepared by the same method, and the Ce_0.93_Mn_0.07_O_2_ catalyst made the CO conversion rate reach 100% at 255 °C. Numerous experimental studies have demonstrated the superior performance of Ce-MnO_x_ composite oxide in the field of CO catalysts. It can be efficiently applied to reduce catalyst costs and prevent heating tail gas to meet reaction requirements in low-temperature CO catalytic oxidation. At the same time, the subsequent denitrification reactions benefit from temperature compensation provided by the heat generated during the CO catalytic oxidation reaction, greatly reduce energy consumption, and have great research significance.

Considering the previous studies, it is possible to synthesize effective cerium manganese CO catalysts via a couple of methods, but the impact of the cerium manganese ratio on catalytic performance is disregarded. The regulation of the cerium manganese ratio could alter the structure and morphology of the catalyst, thereby improving its performance. In order to investigate the influence of ratios on catalysts, the coprecipitation method, which has the advantages of a simple preparation process, low cost, and easy control of preparation conditions, is adopted.

In the early stage, catalysts such as Ce and Mn were considered one of the effective methods for reducing NO_x_ [19,20]. However, Ce-Mn-O system catalysts have recently demonstrated exceptional efficacy in reducing NO, CO, and other pollutants. As a result, in this paper, Ce-MnO_x_ composite oxide catalysts with different molar ratios are prepared using the coprecipitation method, and the influence of Ce-MnO_x_ catalysts with different molar ratios on the catalytic performance of CO is studied, which showed better conversion rate with lower temperature required. XRD, EDS, SEM, H_2_-TPR, CO-TPD, and in situ infrared detection are employed to characterize the physical and chemical properties of the catalyst. CO-conversion rates achieved 91.98% at 100 °C and 99.96% at 140 °C, which resulted in a considerable decrease in the required reaction temperature as compared to the prior Ce-MnO_x_ composite oxide catalyst.

## 2. Experimental Method

### 2.1. Experimental Reagent

Source of Ce: Ce(NO_3_)_3_·6H_2_O (99% purity), source of Mn: MnC_4_H_6_O_4_·4H_2_O (99% purity), and precipitating agent: NH_3_·H_2_O (25% purity) were purchased from Macklin Inc., Shanghai, China. N_2_ (99.9% concentration), CO (2% concentration), and O_2_ (99.9% concentration) were purchased from Changsha Fanggang Gas Co., Ltd. Changsha, China.

### 2.2. Preparation of CO Oxidation Catalyst

The Ce-MnO_x_ composite oxide catalyst was synthesized via coprecipitation method. A certain amount of cerium nitrate hexahydrate and manganese acetate tetrahydrate were dissolved in deionized water using a mass ratio of Ce:Mn = X:1 (where X = 3, 2, 1, 1/2, 1/3). After 1 h of magnetic stirring, ammonia was slowly added drop by drop at a constant rate of 10 s/drop until the solution pH reached 10. After 2 h of continuous stirring, the solution was allowed to stand for another 10 h until sediment occurred. The sediment was subjected to several washes with suction filtration and deionized water until the pH reached 7, and the precursor was obtained. The precursor was then dried in a blast oven for 5 h at 120 °C and calcined for 4 h at 400 °C in a muffle furnace with a heating rate of 5 °C/min. After that, the prepared catalyst was then cooled naturally to room temperature. Finally, the sample was sieved with 40–60 mesh, and the screened particles were selected for catalyst activity test.

### 2.3. Catalyst Activity Test

In order to test the catalyst activity, a micro-fixed bed reactor was utilized. Ce-MnO_x_ composite oxide catalyst particles were measured using a 5 mL cylinder, and a gas space velocity of 30,000 h^−1^ was set. N_2_ serves as the equilibrium gas in the mixed gas, which also contains an O_2_ concentration of 16% and CO concentration of 6000 ppm. The DX4000 flue gas analyzer manufactured by Gasmet Technologies Oy, Vantaa, Finland was utilized to analyze CO concentration before and after the reaction in real time. The performance of catalyst was examined between 60 °C and 140 °C, with the test temperature being adjusted step by step. The CO conversion rate of the catalyst was calculated by the following formula:

(1) $$\mathrm{CO}\;\mathrm{conversion}\;\mathrm{rate}\;\eta =\frac{{\left[\mathrm{CO}\right]}_{\mathrm{in}}-{\left[\mathrm{CO}\right]}_{\mathrm{out}}}{{\left[\mathrm{CO}\right]}_{\mathrm{in}}}\times 100\%$$

### 2.4. Characterization of Catalysts

The phase analysis was detected by the D8 ADVANCE, Bruker, Karlsruhe, German using a Cu-K_α_ source with a scan rate of 5°/min and a step size of 0.02° and a 2θ angle ranging from 5 to 80°. The structure and element distribution of the sample were examined using Talos F200i, Thermo Fisher Scientific Inc, Massachusetts, America. The specific surface area was determined using Quadasorb SI-3, Anton Paar QuantaTec Inc., Boynton Beach, FL, USA. The surface microstructure was analyzed with JSM-6490LV, JEOL, Tokyo, Japan. Catalytic reduction capacity and surface CO adsorption–desorption were assessed using the Autochem II 2920, Micromeritics Instruments Corporation, Norcross, GA, USA. Detailed CO-TPD procedures were as follows: weigh and load 0.1 g sample into a U-shaped tube filled with quartz cotton. Raise the temperature at a rate of 10 °C/min to 180 °C, and purify the catalyst with constant temperature pre-treatment in He atmosphere for 60 min to remove impurities such as H_2_O and CO_2_. Afterward, after cooling to 50 °C, switch to a 9.84% CO/He mixture gas, and maintain blowing for 60 min to achieve adsorption saturation on the catalyst surface. Then, switch to He gas and maintain for 60 min to remove residual CO in the tube. Finally, continuously introduce He gas and heat up to 900 °C at a heating rate of 10 °C/min under the He atmosphere for testing. CO-DRIFTS was tested on the Nicolet 20, Thermo Fisher Scientific Inc., Waltham, MA, USA. 

## 3. Results and Discussion

### 3.1. Catalyst Performance Evaluation

**Figure 1:**
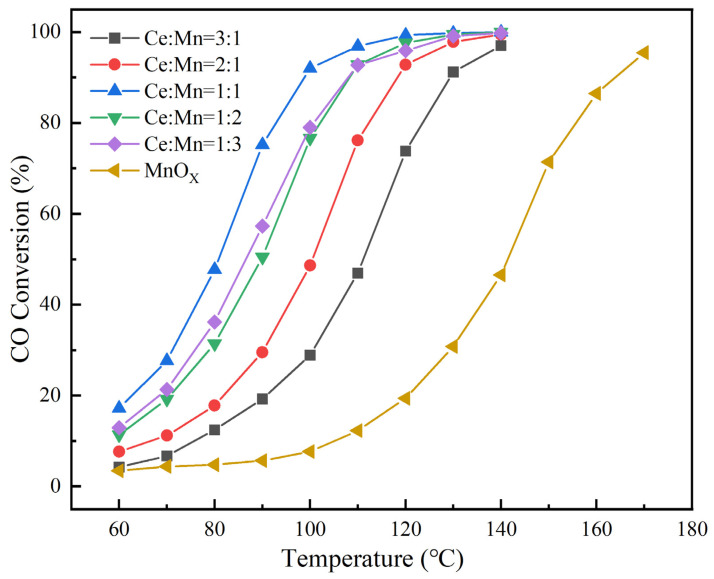
Evaluation results of Ce-MnO_x_ catalyst performance with different proportions.

Figure 1 presents the results of the performance evaluation of Ce-MnO_x_ composite oxide catalysts with varying proportions. As can be seen, the CO conversion efficiency increases with increasing catalyst temperature, while the catalyst performance first increases and then decreases with decreasing Ce content. The catalytic efficiency of a single MnO_x_ catalyst is relatively poor, with a CO conversion efficiency of only 46.57% at 140 °C and 95.4% at 170 °C. However, the introduction of Ce significantly enhances the catalytic performance, drastically reducing the catalytic temperature. All of the Ce-MnO_x_ catalysts achieve CO conversion rates above 96% at 140 °C, and the Ce-MnO_x_ catalyst with the greatest catalytic activity has a Ce:Mn ratio of 1:1. At a temperature of 100 °C, the CO conversion rate reaches 91.98%, while the conversion rate of CO of single MnO_x_ catalyst is only 7.66%. Furthermore, the Ce-MnO_x_ catalyst demonstrates an exceptional CO conversion rate of 99.96% at a higher temperature of 140 °C. Further elaboration on the catalytic mechanism will be discussed in detail later.

### 3.2. XRD and EDS Characterization Results for Catalysts

**Figure 2:**
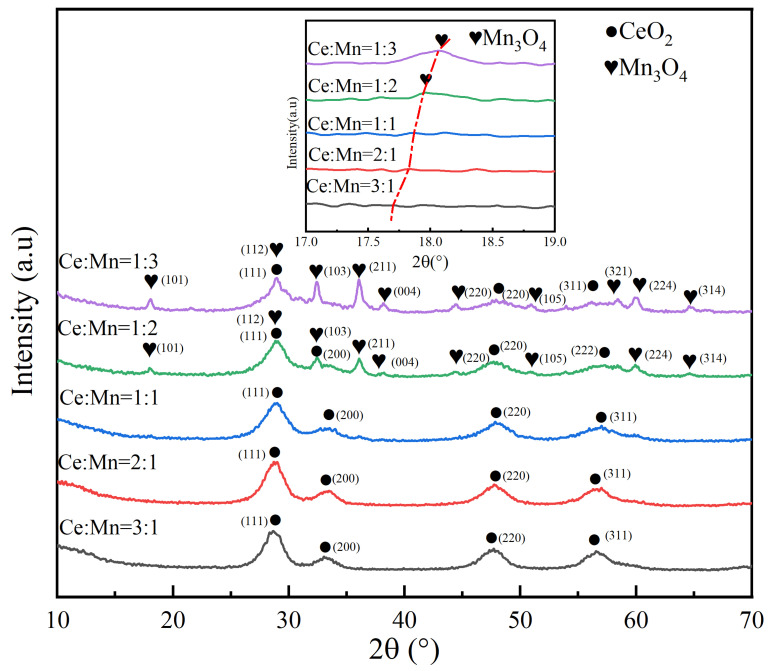
XRD characterization of Ce-MnO_x_ catalysts with different proportions.

The phase analysis of Ce-MnO_x_ composite oxide catalysts in various ratios is illustrated in Figure 2. In the Ce-MnO_x_ catalyst phase, only CeO_2_ can be observed when the Ce:Mn ratio steadily falls from Ce:Mn = 3:1 to Ce:Mn = 1:1, as shown in the figure, leaving the oxide peak of the Mn element undetected. As the Ce content continues to decrease, the Mn_3_O_4_ peak gradually increases, and the CeO_2_ peak gradually decreases. When Ce:Mn = 1:2 and Ce:Mn = 1:3, the main phase of the Ce-MnO_x_ catalyst is Mn_3_O_4_, and the secondary phase is CeO_2_. A clear peak shift phenomenon appears when the phase diagram at 18° is slightly enlarged, indicating that MnO_x_ is mainly highly dispersed and amorphous when the content of Ce is high and has entered the interior of the CeO_2_ lattice [21,22]. Due to the existence of defects and unstable states, amorphous MnO_x_ has a high surface energy that is advantageous for the catalyst reaction since it promotes gas adsorption and reaction [23,24]. As a result, when Ce:Mn = 1:1, the Ce-MnO_x_ catalyst contained a sizable amount of amorphous MnO_x_ that facilitated the quick adsorption and desorption of CO on the catalytic surface, hence increasing CO conversion. The average grain size of test samples was calculated based on the XRD pattern and Scherrer equation, as shown in Table 1. With an increase in Mn content, the average grain size of Mn_3_O_4_ increased from 17.1 nm to 20.5 nm, indicating a rise in the crystallinity of Mn_3_O_4_ in catalysis with the decrease in Ce content. The average grain size of CeO_2_ shows a first decreasing and then increasing trend, and CeO_2_ has the smallest average particle size, measuring only 4.3 nm, when Ce:Mn is 1:1. This is due to the interaction between amorphous Mn_3_O_4_ and CeO_2_, which enables some Mn^x+^ ions to enter the CeO_2_ lattice interior and replace Ce^4+^ ions, thereby forming lattice defects, which decrease the crystallinity of the CeO_2_ lattice and result in a decrease in the average grain size of CeO_2_ [25]. The lower the crystallinity, the more uniformly the material is dispersed, which is beneficial to the improvement in catalyst activity [26].

**Figure 3:**
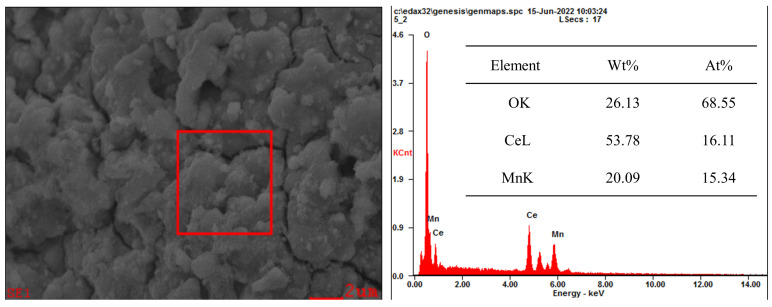
The element distribution results of Ce:Mn = 1:1 catalysts for Ce, Mn.

In order to further observe the element distribution in the Ce-MnO_x_ composite oxide catalyst, the catalyst element proportion with Ce:Mn = 1:1 was characterized by EDS, and the results are displayed in Figure 3. EDS analysis of Ce and Mn was applied to the selected region, and it was found that the atomic ratio of Ce and Mn is close to 1:1, which is in line with experimental expectations, and shows that Ce and Mn elements are evenly distributed in the catalyst. Combined with XRD, the oxide peak of Mn could not be detected in the phase, and it can be inferred that MnO_x_ failed to form a crystal structure, which is mainly amorphous.

### 3.3. TEM Characterization of Catalyst

**Figure 4:**
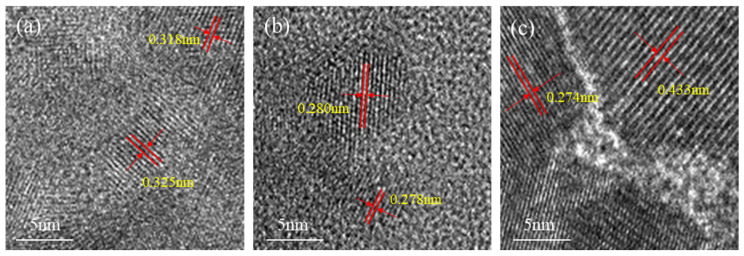
TEM characterization of Ce-MnO_x_ catalysts with different proportions: (**a**) Ce:Mn = 3:1; (**b**) Ce:Mn = 1:1; (**c**) Ce:Mn = 1:3.

TEM characterization was conducted on three Ce-MnO_x_ catalyst samples with different cerium to manganese molar ratios (3:1, 1:1, and 1:3). The results are presented in Figure 4, which demonstrates that lattice spacing of 0.318 nm and 0.325 nm corresponded to the (111) crystal plane of CeO_2_ for a molar ratio of cerium to manganese of 3:1. Cerium–manganese catalysts with a molar ratio of 1:1 have lattice spacing of 0.280 nm and 0.278 nm. In the report of Minakshi et al. [27], because the mean atomic number of CeO_2_ was higher and the defective crystal structure was lessened, they showed stronger diffraction and Kikuchi line contrast than MnO_2_. However, at this ratio, the amorphous Mn^x+^ ion in the Ce-MnO_x_ composite oxide catalyst replaces the Ce^4+^ ion, making the CeO_2_ lattice distorted, resulting in the (111) lattice spacing of CeO_2_ becoming smaller. For a catalyst with a cerium–manganese molar ratio of 1:3, the lattice spacing is 0.274 nm and 0.433 nm. The former corresponds to the (111) crystal plane of the concave CeO_2_, and the latter corresponds to the (112) crystal plane of Mn_3_O_4_. The results of crystal plane analysis are consistent with those of phase analysis results provided in Section 3.2. It is observed that Mn oxide exists in an amorphous state when Ce content is high.

### 3.4. Analysis of the Microscopic Surface Structure of Catalyst

**Figure 5:**
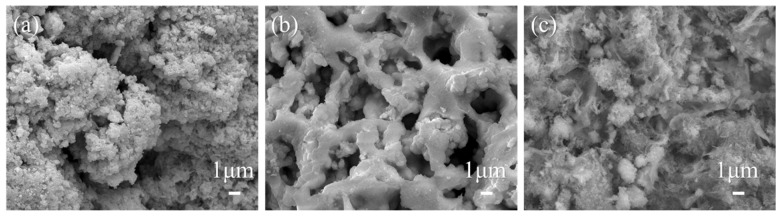
SEM characterization of Ce-MnO_x_ catalysts with different proportions: (**a**) Ce:Mn = 3:1; (**b**) Ce:Mn = 1:1; (**c**) Ce:Mn = 1:3.

The surface morphology and structure of the Ce-MnO_x_ catalyst were analyzed via SEM, as shown in Figure 5. The microstructure of the Ce-MnO_x_ catalyst changes with the decrease of Ce content. Particles build up to form the catalyst at a Ce:Mn ratio of 3:1, resulting in a rough surface with varying pore sizes. When the Ce content is reduced to Ce:Mn = 1:1, the catalyst surface is smooth, and it can be clearly seen that the pore size is similar and evenly distributed. Ce content eventually drops to Ce:Mn = 1:3, at which point the catalyst surface is rough and has a large number of flocculent structures but comparatively few pores and smaller pore sizes. Uniform pores are visible in the morphology of the catalyst with Ce:Mn = 1:1, which results in well-scattered active sites that facilitate gas adsorption and desorption. As a result, the catalyst at a Ce:Mn of 1:1 displays the best performance.

### 3.5. Characterization of Catalyst H_2_ Temperature Programmed Reduction (H_2_-TPR)

**Figure 6:**
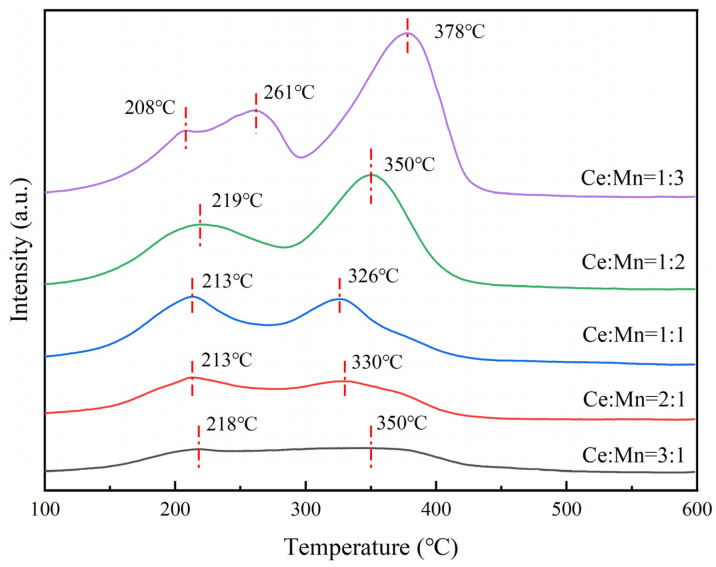
H_2_-TPR characterization of Ce-MnO_x_ catalysts with different proportions.

The redox performance of the Ce-MnO_x_ catalyst was evaluated by H_2_-TPR, and the results are shown in Figure 6. With the exception of Ce:Mn = 1:3, which exhibits three reduction peaks, only two reduction peaks are observed in other component samples for Ce-MnO_x_ catalysts below 600 °C. The generation of highly dispersed MnO_x_ clusters by a limited number of readily reduced manganese species results in a tiny peak at 208 °C when Ce:Mn = 1:3 [28,29]. The reduction of MnO_2_ and Mn_2_O_3_ to Mn_3_O_4_ results in the first reduction peak, which is located at 220 °C; the second reduction peak, which is located at 350 °C, is caused by the reduction of Mn_3_O_4_ to MnO and the reduction of oxygen on the surface of CeO_2_ [30]. As the content of the Ce element decreases from Ce:Mn = 3:1 to Ce:Mn = 1:1, the main reduction peaks of the catalyst all migrate to the low-temperature zone, and the intensity of the corresponding peaks increases. These findings imply that when the cerium concentration decreases, the interaction between cerium and manganese oxides is strengthened, promoting the reduction behavior of cerium and manganese in the sample and improving the fluidity of oxygen species. After that, the content of Ce element continues to decrease, the main reduction peak of the catalyst moves to the high-temperature zone, and the corresponding peak strength increases, and the second reduction peak is more obvious, which also reveals that the two main components of the catalyst in Ce:Mn = 1:2 and Ce:Mn = 1:3 with higher Mn content are Mn_3_O_4_. These findings are also consistent with the XRD phase analysis results found in Section 3.2.

### 3.6. Characterization of Catalyst with CO-Temperature Programmed Desorption (CO-TPD)

**Figure 7:**
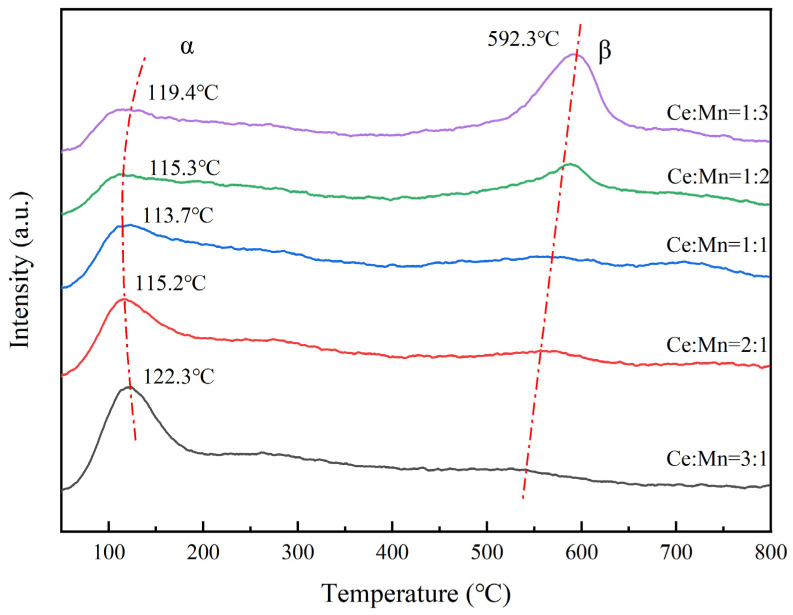
CO-TPD characterization of Ce-MnO_x_ catalysts with different proportions.

Figure 7 displays the process of CO adsorption, heating, and CO_2_ release on the surface of Ce-MnO_x_ composite oxide catalysts in varying proportions. The catalysts exhibit two similar analytical peaks, named α and β peaks, which means that there are two different processes for adsorbing CO and desorbing CO_2_. This may be related to different oxygen species on the catalyst: α peak is the analytical peak of CO and active oxygen on the catalyst surface to generate CO_2_; β peak is the analytical peak of CO and catalyst lattice oxygen to generate CO_2_ [31]. The lowest temperature of 113.7 °C obtained as Ce:Mn is 1:1. A reduction in Ce concentration leads to a low-temperature shift and is followed by a high-temperature shift for the αpeak. Meanwhile, the β peak gradually moves to the high temperature with the decrease in Ce content, and the peak area increases continuously as well. The results reveal that with the decrease in Ce content, the lattice oxygen content gradually increases. The catalytic activity of the catalyst is generally related to the α peak [32]. The lower the α peak temperature, the more conducive to the evolution of CO_2_, and the higher the catalytic activity. It is consistent with the optimum catalytic activity of Ce-MnO_x_ composite oxide catalyst at Ce:Mn of 1:1 that the analytical peak temperature for CO combined with surface active oxygen to generate CO_2_ is the lowest at Ce:Mn = 1:1. This might be due to the abundance of amorphous MnO_x_ exposing more active catalyst sites, increasing the quantity of active oxygen on the surface and considerably enhancing the catalytic performance of the catalyst.

### 3.7. In Situ Infrared Diffuse Reflection Characterization of Catalysts

**Figure 8:**
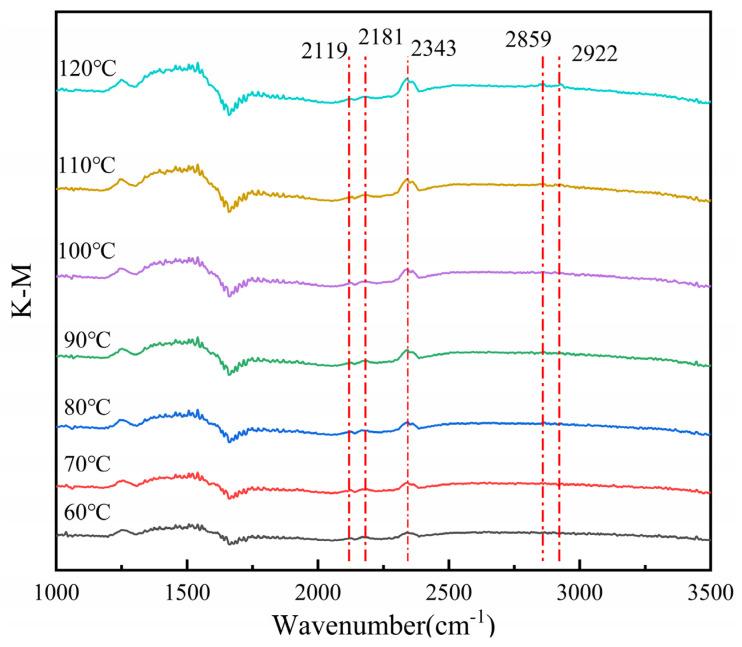
CO-DRIFTS characterization of Ce-MnO_x_ catalysts with Ce:Mn = 1:1.

According to the results from the evaluation of catalytic activity, the Ce-MnO_x_ composite oxide catalyst with Ce:Mn ratio of 1:1, which demonstrated the best performance, was adopted. A sample weighing 0.08 g was selected for testing. The sample was processed for 10 min at 120 °C in a nitrogen-purge environment to remove the residual water. After the treatment was completed, the sample was cooled to room temperature, and a mixed gas (N_2_ used as equilibrium gas, with 16% O_2_ concentration and CO concentration of 6000 ppm) was introduced and gradually heated up from 60 °C to 120 °C. In situ infrared spectroscopy was carried out when the temperature stabilized at each testing temperature, and the results are displayed in Figure 8. The absorption peak at 2343 cm^−1^ in the figure gradually increases with the increase in temperature, which is attributed to CO_2_ [32]. With the increment in temperature, the catalytic activity of the sample increases, and a considerable amount of CO is converted to CO_2_, resulting in an elevation of the peak here. The absorption peaks at 2119 cm^−1^ and 2181 cm^−1^ are CO absorption peaks, which gradually decrease with the increment in temperature [32,33,34]. The absorption peaks of 2895 cm^−1^ and 2922 cm^−1^ are attributed to the stretching vibration of the C-H bond [35]. The absorption peaks from 1200 cm^−1^ to 1800 cm^−1^ are connected to the vibration of carbonate species [35,36,37,38], and peak strength increases with the rising temperature. 

Due to the low temperature at which the CO reaction is catalyzed by the Ce-MnO_x_ catalyst, the L-H mechanism (Langmuir-the Hinshelwood mechanism) should be followed, which has a low activation energy requirement. Combined with in situ infrared spectroscopy and the L-H mechanism, it can be inferred that CO and O_2_ in the mixed gas are adsorbed on the catalyst surface, and the adsorbed [CO] and [O] are activated into active intermediate carbonate in the active center of the catalyst and then decomposed into CO_2_ [39,40].

## 4. Conclusions

(1) The catalytic efficiency of pure-phase MnO_x_ catalyst is 95.4% at 170 °C. While the performance of the Ce-doped catalyst is greatly improved, the Ce-MnO_x_ catalyst can achieve more than 96% CO conversion rate at 140 °C. As the amount of Ce element diminishes, the catalyst performance initially rises and then falls. The best catalyst performance is achieved when the ratio of Ce to Mn is 1:1, where the CO-removal rate can reach 91.98% at 100°C and 99.96% at 140 °C.
(2) The Ce-MnO_x_ catalyst phase contains only CeO_2_ for Ce:Mn ≥ 1, and MnO_x_ exists in an amorphous form; when Ce:Mn < 1, the Mn element mainly exists in the form of Mn_3_O_4_, and the secondary phase is CeO2. For Ce:Mn = 1:1, the Ce-MnO_x_ catalyst has numerous amorphous MnO_x_. When Ce:Mn = 1:1, the Ce-MnOx catalyst has a large amount of amorphous MnOx and possesses excellent characteristics, such as low crystallinity and uniform element and pore distribution. Due to its distinguished reduction performance, CO adsorption, and desorption performance, the composite catalyst exhibits a good CO-conversion rate at lower temperatures.
(3) The Ce-MnO_x_ catalyst follows the L-H mechanism for CO catalysis. The adsorbed [CO] reacts with the adsorbed [O] to generate an active center on the surface of the catalyst and then generates the active intermediate carbonate, which is decomposed to generate CO_2_ subsequently.

## Figures and Tables

**Table 1:** Average grain size of phase of double oxide catalyst.

Ce:Mn	The Average Grain Size/nm	
	CeO_2_	Mn_3_O_4_
3:1	6.5	0
2:1	4.7	0
1:1	4.3	0
1:2	5.0	17.1
1:3	6.3	20.5

## Data Availability

Not applicable.
